# Isolate Identity Determines Plant Tolerance to Pathogen Attack in Assembled Mycorrhizal Communities

**DOI:** 10.1371/journal.pone.0061329

**Published:** 2013-04-19

**Authors:** Thaddeus J. Lewandowski, Kari E. Dunfield, Pedro M. Antunes

**Affiliations:** 1 Invasive Species Research Institute and Biology Department, Algoma University, Sault Ste. Marie, Ontario, Canada; 2 School of Environmental Sciences, University of Guelph, Guelph, Ontario, Canada; University of Tartu, Estonia

## Abstract

Arbuscular mycorrhizal fungi (AMF) are widespread soil microorganisms that associate mutualistically with plant hosts. AMF receive photosynthates from the host in return for various benefits. One of such benefits is in the form of enhanced pathogen tolerance. However, this aspect of the symbiosis has been understudied compared to effects on plant growth and its ability to acquire nutrients. While it is known that increased AMF species richness positively correlates with plant productivity, the relationship between AMF diversity and host responses to pathogen attack remains obscure. The objective of this study was to test whether AMF isolates can differentially attenuate the deleterious effects of a root pathogen on plant growth, whether the richest assemblage of AMF isolates provides the most tolerance against the pathogen, and whether AMF-induced changes to root architecture serve as a mechanism for improved plant disease tolerance. In a growth chamber study, we exposed the plant oxeye daisy (*Leucanthemum vulgare*) to all combinations of three AMF isolates and to the plant root pathogen *Rhizoctonia solani*. We found that the pathogen caused an 81% reduction in shoot and a 70% reduction in root biomass. AMF significantly reduced the highly deleterious effect of the pathogen. Mycorrhizal plants infected with the pathogen produced 91% more dry shoot biomass and 72% more dry root biomass relative to plants solely infected with *R. solani*. AMF isolate identity was a better predictor of AMF-mediated host tolerance to the pathogen than AMF richness. However, the enhanced tolerance response did not result from AMF-mediated changes to root architecture. Our data indicate that AMF communities can play a major role in alleviating host pathogen attack but this depends primarily on the capacity of individual AMF isolates to provide this benefit.

## Introduction

Arbuscular mycorrhizal fungi (AMF) are obligate plant symbionts that appear to have facilitated the establishment of plants in terrestrial systems [1]. Estimates approximate that 80% of land plants associate with AMF [2]. Plant hosts provide the fungal partner with carbohydrates and receive nutrients and other benefits in return [3].

Improved plant nutrition has been the focus of numerous studies of the AMF-plant symbiosis, but AMF also improve host tolerance to pathogen infections [4] even without enhancing plant nutrition [5]. The mechanism(s) by which AMF enhance host tolerance to pathogen attack is (are) not well understood [6]. A recent study found evidence of AMF-mediated host pathogen tolerance by indigenous AMF assemblages and suggested that such an effect is plant species-specific [7], which may be, at least in part, associated with the species' root morphology [8]. On the fungal side of the symbiosis, research on AMF - pathogen interactions has focused primarily on single AMF isolates in defense against a single pathogen [8]–[14]. Overall, these studies indicate that AMF can significantly reduce disease severity, particularly those AMF belonging to the order Glomerales [8], [9].

AMF richness appears to be positively correlated with plant productivity, supporting niche complementarity and synergism within functionally overlapping isolates [15], [16]. As such, it is possible that richer AMF communities offer greater benefit to host plants in the form of pathogen tolerance. This hypothesis has only been partially tested in recent years. A study by Mwangi *et al*. [17] investigated the performance of eight AMF isolates from different taxa in their defense of *Lycopersicon esculentum* against the pathogen *Fusarium oxysporum* f. sp. *lycopersici*. Although the AMF consortium reduced disease severity and increased plant height and root dry weight compared to non-mycorrhizal controls, the study did not consider the individual effects of each AMF isolate that was part of the consortium. Furthermore, pathogen-free experimental groups were excluded from the design, thereby excluding the possibility of testing for potential shifts in mycorrizal responsiveness due to pathogen attack. Jaiti *et al.* [18] investigated the response of *Phoenix dactylifera* against the pathogen *F. oxysporum* f. sp. *albedinis* in the presence of three individual AMF isolates (*Glomus monosporus*, *G. deserticola* and *G. clarum*) and an AMF consortium native to southern Morocco comprised of *Glomus* spp., *Sclerosystis* spp., *Acaulospora* spp. and *Scutellospora* spp. The consortium increased plant survival relative to single AMF isolates after introducing the pathogen. However, it did not consistently outperform single AMF isolates for other plant responses, including shoot height and weight and number of leaves.

Newsham *et al.* [19] suggested that relationships between the mycorrhizal symbiosis and plant pathogens might be related to AMF-induced changes to plant root physiology and architecture. Many studies have found that AMF alter root growth and architecture [20]–[26]. Generally, it is hypothesized that larger effects of AMF on host biomass result in root systems with less but more elongated branches, to access a larger soil volume that mycorrhizae can exploit [20]. However, this aspect was not investigated in studies that incorporated host pathogen-AMF diversity relationships e.g., [17], [18].

Relatively little research has focused on the mechanistic role of AMF to “defend” against pathogen attack through changes in root architecture, and the available data are inconsistent. Norman *et al.* [12] investigated changes to root order and necrosis before and after introducing the pathogen *Phytophthora fragariae* var. *fragariae* to roots of different *Fragaria*×*ananassa* cultivars colonized by two individual AMF isolates of different species. Before pathogen introduction, all but one of six possible combinations of *F.*×*ananassa* cultivar and AMF displayed fifth order branching, while non-AMF plants displayed branching of a smaller magnitude. After the introduction of the pathogen, AMF reduced root necrosis in cultivars that were most susceptible to the pathogen but changes in root architecture varied amongst individual AMF. In contrast to Norman *et al.* [12], Vigo *et al.* [27] did not observe AMF-mediated changes to root architecture in *L. esculentum* prior to introducing the pathogen *P. parasitica*. However, in the presence of the pathogen, roots colonized by AMF increased 50% in length and exhibited a reduction in root necrosis compared to non-colonized roots. In another study, Trotta *et al.* [28] demonstrated that while the AMF *G. mosseae* alleviated the effects of the pathogen *P. nicotianae* var. *parasitica* on the growth and root necrosis of *L. esculentum*, root branching was reduced. In addition, fewer first order lateral roots were found regardless of the presence of the pathogen. Similar to the studies investigating AMF by pathogen interactions, those investigating root architecture responses to the symbiosis have focused solely on single AMF isolates. As plants associate with AMF communities in field soils [29], an investigation of the contribution by multiple AMF isolates would more applicable to natural conditions.

Although there is evidence that multiple rather than single AMF isolates can provide greater alleviation against plant pathogen attack, intermediate levels of diversity have not been considered. Focusing on P uptake, Jansa *et al.* [30] showed that mixtures of any of three AMF isolates, each representing a different species, did not outperform any of the single AMF isolates used in those mixtures. Furthermore, their work indicated that symbiotic effects resulting from the isolate mixtures were primarily due to the action of a more effective isolate. Bennett and Bever [31] tested whether three AMF isolates differentially altered plant host responses to herbivory. They found that one of the three isolates acted as a “super fungus” driving those responses. There have not been studies to determine if other AMF benefits such as enhanced host tolerance to pathogen attack follow similar trends. The questions of how a plant's root architecture responds to AMF diversity in the presence of a pathogen and the relationship between those responses and putative changes in pathogen tolerance have also not been investigated.

While the study of plant pathogen defense by AMF has primarily focused on economically important crop species (see above), the interaction between AMF and pathogens is also relevant in the study of natural systems. For instance, enhanced plant pathogen tolerance may play an important role in the establishment of exotic plant species in communities. Exotic invaders accumulate at least generalist pathogens [32], [33], but may exploit soil mutualisms to their advantage in the invaded range [34]. Research on how AMF diversity may contribute to improved disease tolerance in exotic species is critical to understanding plant invasions and could provide valuable information for risk assessment.

In this study we aimed to address the following questions: 1) Do AMF isolates differentially attenuate the deleterious effects of a root pathogen on plant growth?, 2) Does the richest assemblage of AMF isolates provide the greatest tolerance to the pathogen?, and 3) Do AMF-induced changes to the host's root architecture serve as a mechanism for improved plant disease tolerance?

## Materials and Methods

### Substrate and growth conditions

Field soil was collected from the Invasive Species Research Institute's long-term research field site at the Ontario Forestry Research Institute arboretum in Sault Ste. Marie, ON, Canada in October 2011 (N 46°32.574′, W 84°27.543′). This silty loam soil had a pH of 5.8 and contained 0.13% total N, 1.81% total C, 11.4 mg plant extractable P kg^−1^, 360.7 mg Ca kg^−1^, 48.9 mg K kg^−1^, and 38.9 Mg kg^−1^. The soil was sterilized (i.e., autoclaved twice for one hour at 121°C in a vacuum cycle) and stored at room temperature for 19 days to allow mineralization by incoming airborne bacterial communities. The soil was then mixed in equal ratios with sand (non-calcareous “B” sand, Hutcheson Sand and Mixes, Huntsville, ON, CA) and Turface (montmorillonite clay, Turface Athletics MVP, Profile Products LLC, Buffalo Grove, IL, USA) to create a bulk substrate free of viable AMF. The experiment was carried out in a plant growth chamber set to 70% relative humidity, 16 hours of light at an intensity of 130 µmol m^−2^ s^−1^ at 22°C and eight hours of dark at 15°C. At the beginning of the experiment, 151 ml containers (Ray Leach Cone-tainers; Stuewe and Sons Inc., Corvallis, OR, USA) were cleaned and surface-sterilized in 10% bleach for 10 minutes before being filled by the substrate described above. Plants were subsequently transferred to sterile three-litre pots (see below).

### Study organisms

We selected *Leucanthemum vulgare* Lam. (syn. *Chrysanthemum leucanthemum* L., “ox-eye daisy”), which is a perennial member of the Asteraceae Family, as a test plant. *L. vulgare* is mycorrhizal [35], native to Europe and strongly invasive across North American plant communities where it can replace up to 50% of grass in a pasture [36]. Seeds were purchased from the American Meadows Seed Company (Williston, VT, USA) in November of 2010.

AMF inocula isolated in North America (Minnesota, USA) were obtained from the International Culture Collection of Arbuscular Mycorrhizal Fungi (INVAM, West Virginia, USA; http://invam.caf.wvu.edu/index.html). Inocula were examined to confirm that healthy-looking spores were abundant and that no spores of non-target species were present. The selected AMF isolates and their associated INVAM accession numbers were *Glomus intraradices* N.C. Schenk and G.S. Sm. (MN 502), *G. clarum* T.H. Nicolson and N.C. Schenk (MN 414B) and *G. etunicatum* W. N. Becker and Gerd. (MN 501). These species have recently been renamed *Rhizophagus intraradices*, *Rhizophagus clarus* and *Claroideoglomus etunicatum*, respectively [37].

A strain of the fungal root pathogen *Rhizoctonia solani* Kuhn isolated from milkweed (*Asclepias syriaca* L.) growing in Ontario was obtained from Professor Greg Boland of the Pathology Laboratory at the University of Guelph. Strains of *R. solani* are known to affect several species of plants globally by means of seed rot, hypocotyl rot, aerial and web blights, canker, crown rot and root rot [38]–[40]. The strain of *R. solani* used in this study was confirmed to have a pathogenic effect on *L. vulgare* by significantly reducing root length and total biomass (data not shown).

### Experimental design

The experiment was arranged in a completely randomized design consisting of the crossed factors ‘AMF’ and ‘Pathogen’. Specifically, ‘AMF’ included a non-mycorrhizal control treatment and AMF treatments in all possible combinations for a total of eight treatment groups, each of which were grown in the presence or absence of the root pathogen. Each treatment combination was replicated 11 times for a total of 176 experimental units. One pot per treatment was originally prepared with the intent of determining the status of AMF colonization through destructive sampling during the term of the experiment. However, only the non-mycorrhizal control, *R. intraradices* and tri-level AMF isolate treatments inoculated with the pathogen were destructively sampled for this purpose.

Seeds of *L. vulgare* were pre-germinated in moist vermiculite in the growth chamber and seedlings emerged three days after planting. To inoculate plant roots with AMF, an empty sterile borosilicate test tube was inserted three cm into the sterile bulk substrate of the container and the substrate was moistened. Upon removal of the test tube, a cavity was created into which six ml of inoculum of all combinations of AMF was deposited. The subsequent transplant of the seedling into the cavity (four days after emergence) allowed the root system to be in direct contact with the inoculum to increase the chances of successful AMF colonization. For the control treatment, six ml of sterile inoculum substrate was added to the containers, and treatments with increasing levels of diversity received equal volumes of each AMF isolate inoculant. To standardize for non-mycorrhizal microbes, five ml of a microbial wash was prepared from combined inoculums of each AMF isolate and added to all replicates one day after transplant [41], [42].

Two weeks after transplanting, one replicate from each treatment was destructively harvested to confirm AMF root colonization following the methods of Brundrett [43]. With the exceptions of the control and *R. clarus* treatments, AMF colonization was observed in all treatments.

To introduce the pathogen, inoculum consisting of ground *Lolium perenne* seeds heavily infected with *R. solani* was mixed by hand at a density of 5 g L^−1^ into the sterile substrate (non-pathogen controls received the same amount of sterilized rye grass inoculum; 20 min at 121°C). Similar to the procedure to transplant seedlings from vermiculite using borosilicate test tubes, an empty sterile container was depressed into the pathogen substrate at the centre of a three-litre pot and wetted. The cavity created by the removal of the empty container formed the exact shape to insert each “plug” consisting of each five-week-old plant with its root system and associated AMF treatment. This method ensured that further plant root growth had to pass through the pathogen inoculum. Experimental units were re-randomized at the time of pathogen introduction and plants were grown for an additional five weeks. The soil was not fertilized and the pots were watered to field capacity every other day until the end of the ninth week. Watering was interrupted during the final week of the experiment to stimulate AMF sporulation [44], [45].

### Response variables

Plants were destructively harvested after 10 weeks of growth. Substrate was removed from plant root systems and dried at room temperature in unsealed plastic storage bags. Shoots and roots were separated at the soil line; shoots were dried over five days to a constant mass at 60°C and weighed (Mettler Toledo, Richmond Hill, ON, CA). Root systems were stored in 70% ethanol.

All root systems were scanned using the WinRhizo Pro (2009) scanning software (Regent Instruments Canada Inc.) system with the Epson Expression 10000 XL scanner. To scan, roots were removed from 70% ethanol and immersed in water in a transparent tray provided by Regent Instruments. Data were collected for each replicate for the following response variables: total root length, surface area, average diameter, volume occupied in soil, number of forks and number of tips. After scanning, roots were dried at 60°C to a constant mass over five days and weighed.

After drying, root systems of four randomly selected replicates from the control group and each single isolate level of AMF diversity for both pathogen and non-pathogen treatments were rehydrated for 24 hours in deionized water to quantify AMF root colonization following the same staining method cited above [43]. AMF colonization was quantified following the grid-line intersect method of McGonigle [46]. Mycorrhizal structures were not found in the non-mycorrhizal groups. We observed an abundance of *R. solani* hyphae (i.e. with morphology consistent with that described by Parmeter [40]) predominantly around the root tips of plants of the pathogen-inoculated treatments.

For dual- and tri-level AMF isolate assemblages, 50–100 g subsamples of substrate from three randomly selected replicates from both the pathogen and non-pathogen treatments were chosen to morphologically identify spores of each AMF species following the sucrose-suspension method of Brundrett [43]. In all of the selected subsamples, spores of each isolate from the respective treatment group were confirmed, thus supporting the contribution of each individual isolate when in combination with other isolates. No spores were present in non-mycorrhizal controls.

### Statistical analysis

Data on the percentage of root length colonized by AMF were first analysed by a fully crossed two-way MANOVA with ‘AMF’ and ‘Pathogen’ as main factors. AMF response variables (i.e., percent of root length colonized by hyphae, arbuscules and vesicules) were arcsin transformed to protect against violations of the test's assumptions of homoscedasticity and normality. The MANOVA was followed by two-way ANOVAs on each of the three dependent variables and subsequent post-hoc tests were conducted using Tukey's HSD.

We performed individual fully crossed two-factor ANOVAs on shoot and root dry biomass, which were highly correlated (Pearson = 0.90, P<0.01) and, as such, unsuitable for MANOVA [47]. We also performed an ANOVA for shoot to root ratio. Data were box-cox transformed to protect against violation of homoscedasticity and normality of the ANOVA. Since an ‘AMF’ by ‘Pathogen’ interaction was detected, we followed up with post-hoc tests and pre-planned orthogonal contrasts. To address the first question we first determined whether each non-mycorrhizal and pathogen control differed from their respective collective AMF treatments. In addition, post-hoc analyses of plant growth responses among mycorrhizal treatments, including non-mycorrhizal controls, were conducted using Tukey's HSD. To address the second question, follow up contrasts tested whether (one and two), and (two and three) AMF isolate assemblages differed when the pathogen was present. In regard to the third question, all root measurements (i.e., dry root biomass, root length, root surface area, average root diameter, root volume, root tips and forks) were highly correlated with one another (0.701>Pearson<0.994, *p*<0.0001). As such, for reference, only a two-way ANOVA on box cox transformed dry root biomass data is reported. All statistical procedures were performed using STATISTICA data analysis software (StatSoft, Inc., 2011, version 10. www.statsoft.com) and graphed using SigmaPlot graphing software (Systat Software, Inc., 2012, version 12, San Jose CA, USA).

## Results

All AMF isolates colonized *L. vulgare* regardless of the presence of the root pathogen *R. solani*. The MANOVA on all three structures associated with intra-radical AMF root colonization (i.e., hyphae, arbuscules and vesicules) indicated that colonization significantly varied with AMF isolate identity (Table 1). Generally, *R. intraradices* and *R. clarus* colonized the roots more profusely than *C. etunicatum*. The MANOVA also indicated that the presence of the pathogen significantly increased AMF colonization, primarily the number of arbuscules. A significant AMF by pathogen interaction was not detected in the MANOVA, indicating that the pathogen did not change the relative abundances of intra-radical structures produced by the three AMF isolates (Tables 1 and 2).

**Table 1:** Percentage of root length colonized by each AMF isolate at the single diversity level, either in the presence or absence of the pathogen *R. solani*.

	Hyphae		Arbuscules		Vesicles	
AMF isolate	Non-Pathogen	Pathogen	Non-Pathogen	Pathogen	Non-Pathogen	Pathogen
*R. intraradices*	11.1±4.90	27.9±8.83	0.4±0.26	7.8±2.62	2.2±0.91	12.6±5.27
*R. clarus*	33.0±6.41	29.5±8.27	3.8±1.86	12.5±5.66	2.6±0.47	3.1±1.76
*C. etunicatum*	2.8±2.83	0.9±0.66	0.4±0.43	0.5±0.27	ND	ND

Values represent the mean (n = 4) ± SE; ND – not detected.

**Table 2:** (M)ANOVA of percent root length colonized by each AMF isolate.

	MANOVA			Hyphae		Arbuscules		Vesicles	
Treatment	*F*	Effect df	Error df	df	*F*	df	*F*	df	*F*
AMF	9.06	6	32 ***	2	18.84 ***	2	6.83 *	2	12.66 **
Path	4.08	3	16 *	1	0.60	1	8.59 *	1	2.86
AMF×Path	1.59	6	32	2	1.56	2	1.82	2	3.56 *
Residual		18	0.03	18	0.01	18	0.01		

Asterisks represent significant differences as calculated by (M)ANOVA (*p<0.05, **p<0.01, ***p<0.001).

The residual mean squares of the ANOVA models are shown in the bottom row.

**Figure 1:**
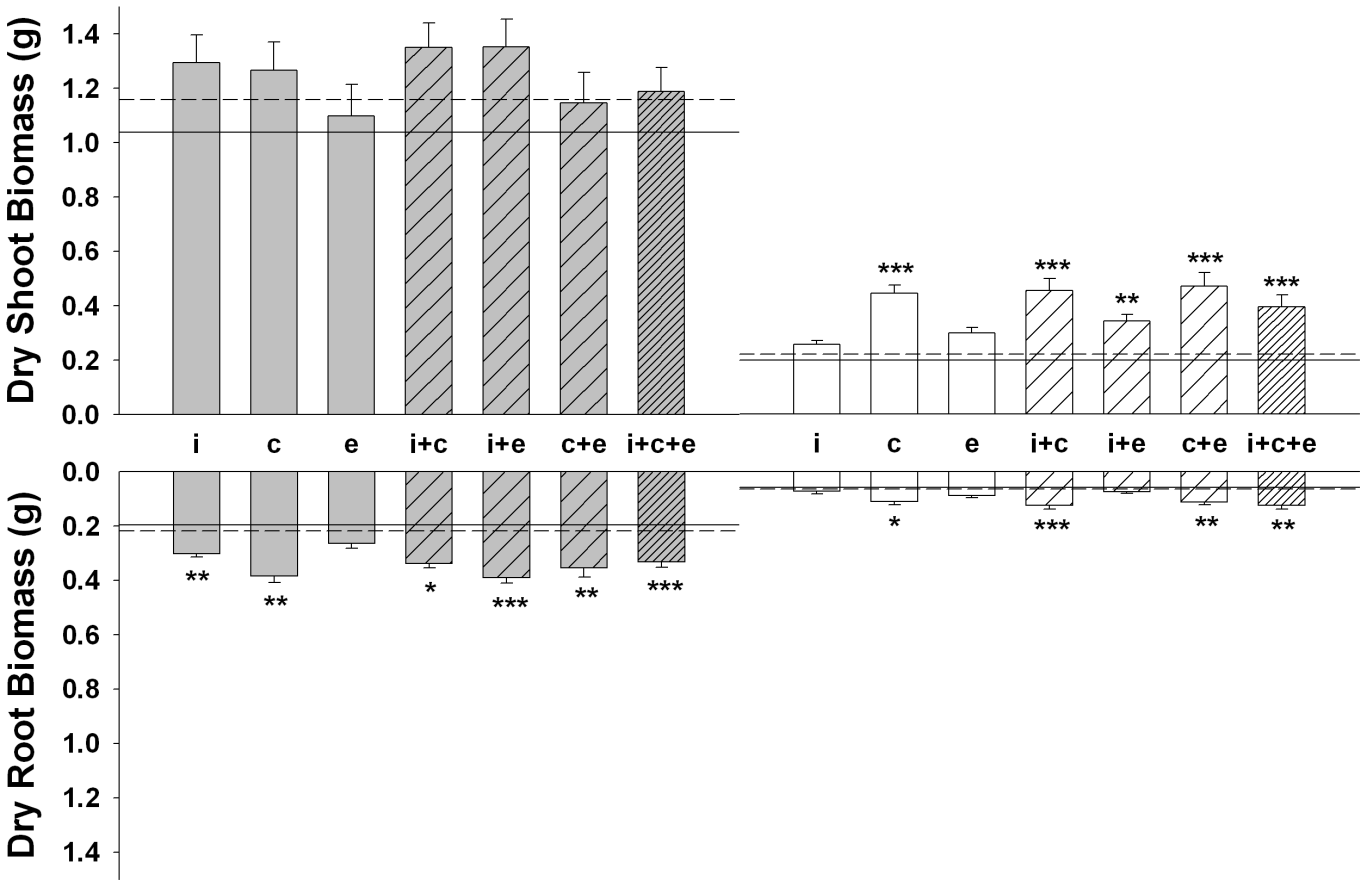
Effect of different AMF assemblages and *R. solani* on the dry shoot and root biomass of *L. vulgare*. Each grey and white bar represents the treatment mean ±1 SE either in absence or presence of *R. solani*, respectively. Open, lighter and heavier diagonal patterns correspond to: single (i.e., i – *R. intraradices*/c – *R. clarus*/e - *C. etunicatum*), dual or tri isolate assemblages, respectively. Horizontal solid and dashed lines correspond to the control mean ±1 SE, respectively. Those over the grey bars correspond to the negative control whereas those over the white bars correspond to the pathogen only (i.e., *R. solani* alone) control. Asterisks represent significant differences to the control calculated by Tukey's HSD (*p<0.05, **p<0.01, ***p<0.001).

The shoot and root biomass of *L. vulgare* were significantly affected by the AMF and root pathogen treatments and we detected a significant interaction between these two factors (Table 3 and Fig. 1). Overall, in absence of the pathogen, *L. vulgare* produced on average 19% more shoot and 73% more root dry biomass in association with AMF relative to the non-mycorrhizal control (Contrast for dry shoot biomass - *F_1,149_* = 5.26, *p* = 0.023; Contrast for dry root biomass - *F_1,149_* = 49.7, *p* = 0.0001) (Fig. 1). However, individually, only inoculation with *R. intraradices*, *R. clarus* and all AMF mixtures significantly promoted root growth in absence of the pathogen (Fig. 1). When considering plant performance by level of AMF isolate assemblage (i.e., isolates combined into either the single, dual or tri level) dry root biomass increased between the single and dual levels (Contrast - *F_1,149_* = 6.01, *p* = 0.015) but not shoot biomass (Contrast - *F_1,149_* = 0.67, *p* = 0.414). In addition, the tri-level isolate mixture did not lead to significantly larger plant biomass than treatments of lower isolate richness.

**Table 3:** ANOVAs of shoot and root biomass.

	Shoot Biomass		Root Biomass		Shoot to Root ratio	
Treatment	df	*F*	df	*F*	df	*F*
AMF	7	7.17 ***	7	12.90 **	7	1.16
Pathogen	1	709.80 ***	1	797.86 ***	1	0.26
AMF×Path	7	3.10 **	7	3.45 **	7	4.64***
Residual	149	0.081	149	0.019	149	0.039

Asterisks represent significant differences as calculated by ANOVA (*p<0.05, **p<0.001, ***p<0.001). The residual mean squares of the ANOVA models are shown in the bottom row.

Relative to the non-mycorrhizal control, the presence of the pathogen alone caused an 81% reduction in shoot (*p*<0.0001) and a 70% reduction in dry root biomass (*p*<0.0001). AMF significantly reduced this highly significant deleterious effect of the pathogen. Overall, mycorrhizal plants infected with the pathogen produced 91% more dry shoot biomass (Contrast - *F_1,149_* = 31.63, *p* = 0.0001) and 72% more dry root biomass (Contrast - *F_1,149_* = 15.67, *p* = 0.0001) than those solely infected with the pathogen (Fig. 1).

The identity and assembly of AMF isolates had a significant effect on the degree to which the symbiosis reduced the deleterious effects of the pathogen. When considering plant performance by level of AMF isolate assemblage (i.e., isolates combined into either the single, dual or tri level) in presence of the pathogen, dry shoot biomass significantly increased from the single to dual levels (Contrast – F*_1,149_* = 8.26, *p*<0.005). This positive diversity-productivity relationship was marginally significant for dry root biomass (Contrast - F*_1,149_* = 2.25, *p*<0.09). The presence of a third isolate did not have an incremental effect on biomass relative to the single or dual isolate mixtures. Individually, *R. intraradices* and *C. etunicatum* did not significantly reduce the deleterious effects of the pathogen, just *R. clarus* did. In fact, the deleterious effects of the pathogen were consistently significantly reduced in mixtures containing *R. clarus*. Although *R. intraradices* and *C. etunicatum* did not alleviate the effects of the pathogen individually, their mixture did have a significant effect in terms of increasing dry shoot biomass relative to the pathogen control (Fig. 1).

**Figure 2:**
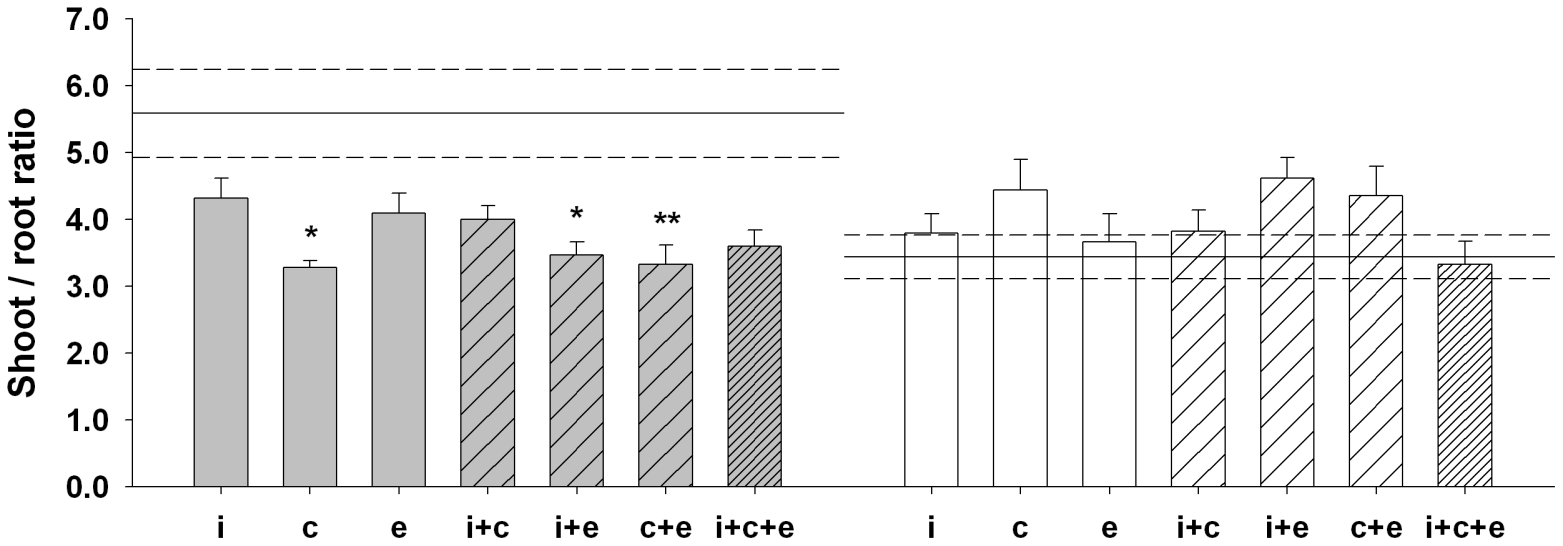
Effect of different AMF assemblages on the shoot to root ratio of *L. vulgare*. Each grey and white bar represents the treatment mean ±1 SE either in absence or presence of *R. solani*, respectively. Open, lighter and heavier diagonal patterns correspond to: single (i.e., i – *R. intraradices*/c – *R. clarus*/e - *C. etunicatum*), dual or tri isolate assemblages, respectively. Horizontal solid and dashed lines correspond to the control mean ±1 SE, respectively. Those over the grey bars correspond to the negative control whereas those over the white bars correspond to the pathogen only (i.e., *R. solani* alone) control. Asterisks represent significant differences to the control calculated by Tukey's HSD (*p<0.05, **p<0.01, ***p<0.001).

All measures of root architecture were significantly correlated with one another and with dry shoot biomass, indicating that ‘AMF’ by ‘Pathogen’ interactions were not modulated by changes in root architecture (only data of dry root biomass are shown). We detected a significant ‘AMF’ by ‘Pathogen’ interaction on shoot to root ratio (Table 3, Fig. 2). AMF significantly enhanced root rather than shoot biomass in absence of the pathogen. In its presence, the shoot to root ratio significantly decreased. However, AMF significantly contributed to alleviate this effect by stimulating relatively more shoot than root biomass (Fig. 2).

## Discussion

*L. vulgare* was colonized by each of the selected AMF isolates and the native strain of *R. solani* had a significant deleterious effect on the plant. This allowed us to address our questions, finding that 1) AMF reduced the deleterious effect caused by a root pathogen, 2) the most AMF-rich assemblage did not best mitigate the deleterious effects of the pathogen (rather, AMF isolate identity was key), and 3) the observed alleviation of the pathogenic effect enabled by the symbiosis did not appear to result from AMF-induced changes to root architecture but rather by reallocation of resources between the shoot and root components of the plant.

In terms of AMF response, the presence of *R. solani* affected AMF colonization of *L. vulgare* by increasing the number of arbuscules. This is in contrast with the results of Abdalla and Abdel-Fattah [48], who found that *R. solani* reduced the number of arbuscules in the roots *Arachis hypogaea*. Conversely, Yao *et al.* [49] reported that *R. solani* had no effect on the colonization of *Solanum tuberosum* by two AMF isolates each of a different species. The increased production of arbuscules may be a stress response, as arbuscules are the site of nutrient exchange between plant and fungal symbionts [3]. Since arbuscules are a sign of vitality, it is possible that the plant is allocating larger amounts of carbon to the roots, thereby stimulating AMF to provide more nutrients and possibly stimulating AMF to trigger other mechanisms in the presence of a pathogen [6].

For plant responses, compared to non-mycorrhizal controls, *R. clarus* promoted plant growth to a greater extent than the other individual AMF isolates regardless of the presence of the pathogen. Importantly, the relative symbiotic performance of each individual AMF isolate was maintained in the different AMF assembly treatments. For instance, any assemblages including *R. clarus* consistently promoted the highest plant productivity. These results are consistent with the idea of sampling effect driven by “super fungi” of greater inherent productivity as proposed by Wardle [50], and indicate that simply increasing AMF richness may not necessarily enhance plant performance synergistically; it is rather the relative contributions of each isolate individually that are maintained as diverse AMF assemblages are constructed. Consistently, in a study focusing on P uptake, Jansa *et al.* [30] observed that plant responses to AMF mixtures were similar to responses to the single AMF isolates used to compose those mixtures, and that a dominant single AMF isolate influenced responses as assemblages were constructed. Also consistently, in a study similarly focusing on multi-trophic interactions between different AMF isolates and aboveground insect herbivory, alleviation of the insect's effect on plant growth was driven primarily by a single *Glomus* isolate [31].

As a result of the hypothesis by Newsham *et al.* [19] (but see also [20]) we identified the need to test the degree to which improved tolerance against a root pathogen provided by AMF could be due to changes in root architecture. We found that, regardless of the presence of the pathogen, AMF had similar proportional effects across all root growth responses. This suggests that, at least in some situations, changes to root architecture may not contribute as much to AMF-mediated pathogen defense as other mechanisms do [6], [20], [51]. We detected a shift in the relative allocation of growth benefits from roots to shoots when the pathogen was present. Once again this effect was stronger for *R. clarus* than for the other isolates, indicating that a more effective mutualist can change the host's relative allocations of carbon to shoots or roots.

Our study included a widespread exotic plant associating with a native fungal pathogen. In spite of the artificial conditions associated with manipulative growth chamber experiments, including the fact that *R. solani* was heavily “bioaugmented”, if we consider the enemy release hypothesis, it is interesting that a generalist pathogen isolated from a native plant host was capable of causing such significant plant growth reductions, nearly 80% less total biomass. On one hand this raises questions about the potential for using native pathogens as potential biological control agents. On the other hand, the alleviation of pathogenic effects enabled by AMF, albeit weak relative to the major negative effect of the pathogen, raises questions about the extent to which the mycorrhizal symbiosis can contribute to promote plant invasions in natural conditions. This idea of exotic invasive plants putatively experiencing positive effects from soil mutualists in introduced ranges hinges on the enhanced mutualisms hypothesis [52], [53], which certainly requires further testing in the context of AMF-mediated alleviation of pathogen effects in natural communities.

Since different AMF isolate assemblages led to different plant responses, our results show that AMF isolate composition is important in the context of host pathogen tolerance. However, AMF species richness alone might not be the most relevant factor when considering plant tolerance to pathogen attack; species and/or isolate identity may play the larger role. Association with certain AMF can be especially critical when plant hosts are stressed by root pathogens, and it is clear that AMF can reduce the negative effect of pathogen attack. Since the interaction between diverse communities of AMF and pathogens is fundamental to understanding how plant communities are assembled, future work should focus on the dynamics of key functional groups (i.e., mutualists and pathogens) that make up the microbial communities present in the roots of plant species in the field.
